# Negative index metamaterial through multi-wave interactions: numerical proof of the concept of low-frequency Lamb-wave multiplexing

**DOI:** 10.1038/s41598-020-79572-9

**Published:** 2021-01-12

**Authors:** Martin Lott, Philippe Roux, Matthieu Rupin, Daniel Colquitt, Andrea Colombi

**Affiliations:** 1 CNRS, IRD, IFSTTAR, ISTerre, University Grenoble Alpes, University Savoie Mont Blanc, 38000 Grenoble, France; 2 CIME Nanotech, Hap2U, Grenoble, France; 3 Department of Mathematical Sciences, University of Liverpool, Liverpool, L69 7ZL UK; 4 Department of Civil, Environmental and Geomatic Engineering, Institute of Structural Engineering, Swiss Federal Institute of Technology, Zurich, Switzerland

**Keywords:** Engineering, Materials for devices, Mechanical properties

## Abstract

We study numerically the potential of a multimodal elastic metamaterial to filter and guide Lamb waves in a plate. Using a sub-wavelength array of elongated beams attached to the plate, and combining the coupling effects of the longitudinal and flexural motion of these resonators, we create narrow transmission bands at the flexural resonances of the beams inside the wide frequency bandgap induced by their longitudinal resonance. The diameter of the beams becomes the tuning parameter for selection of the flexural leakage frequency, without affecting the main bandgap. Finally, by combination of the monopolar and dipolar scattering effects associated with the coupled beam and plate system, we create a frequency-based multiplexer waveguide in a locally resonant metamaterial.

## Introduction

Recent advances in elastic metamaterial design have demonstrated the potential of such metamaterials for the control of wave flow through tuning their mechanical properties at the sub-wavelength scale. Many devices have been successfully tested so far, like lenses^1–3^ or waveguides^4–6^, which have all been based on different physical principles. For example, focusing across a slab can be achieved due to anisotropy and spectral overlap^7–9^, gradient index lenses^3^, and coupled resonant modes^10,11^. Similarly, wave-guides in metamaterials can be obtained through topological insulation techniques^12^ and nonlinear harmonics migration^13^, and even simpler, with defect-like lines^14,15^.

In the following, we present a combination of these physical principles to build a particular elastic waveguide; a multiplexer that can spatially filter an incident plane wave into different point-like sources for the A_0_ Lamb mode. Such multiplexing is made possible through the overlap of two resonant modes of the unit cell of our metamaterial. The metamaterial is constituted of elongated beams attached to a plate, which can couple with the first antisymmetric A_0_ Lamb mode with two types of motions, one longitudinal and one flexural.

**Figure 1:**
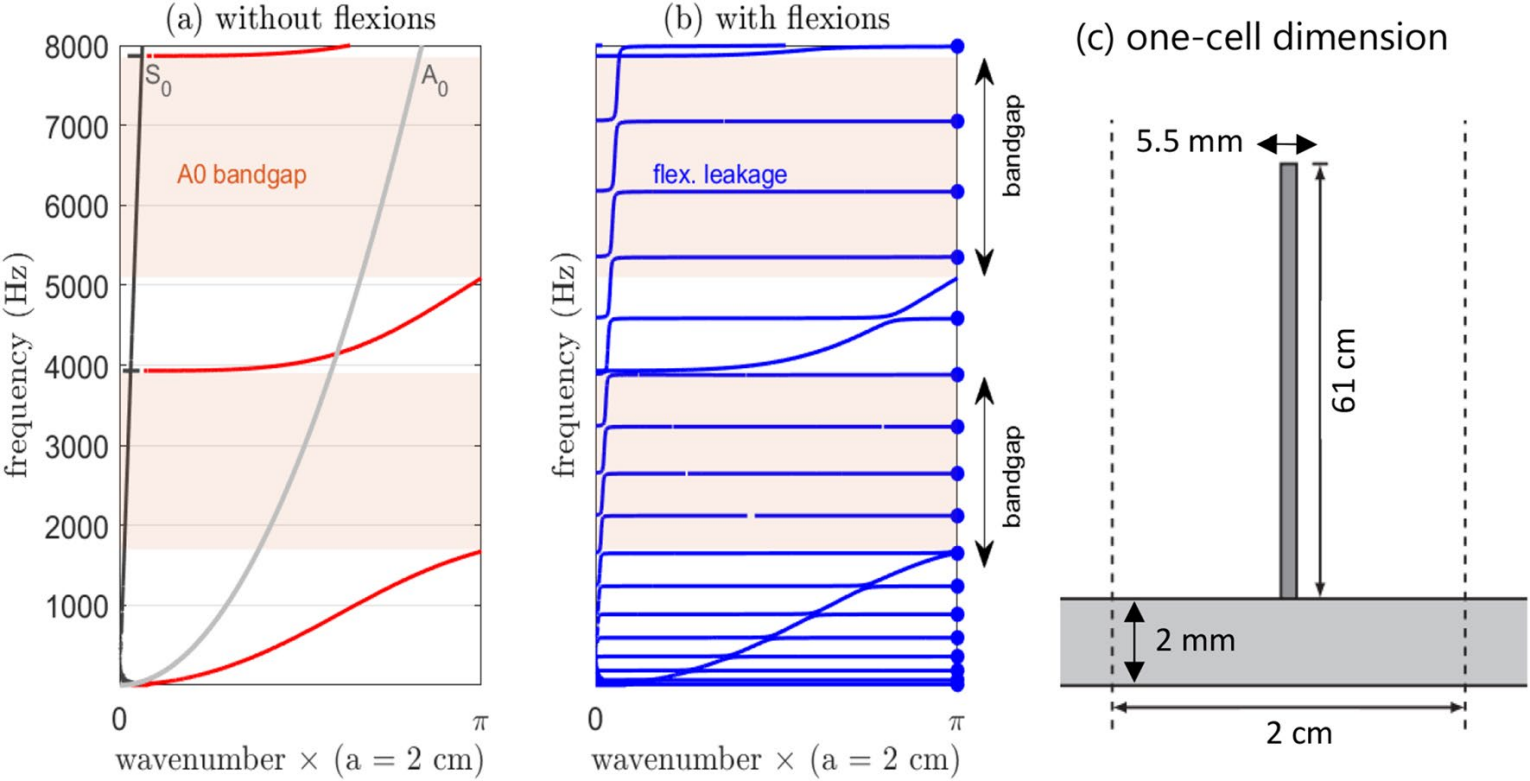
Theoretical dispersion relation for an infinite array of beams [Colquitt 2016]. (**a**) Dispersion curves obtained ignoring beam flexural resonance effects. (**b**) Dispersion curves with flexural resonance effects. (**c**) Cell dimensions and properties: Lattice constant a = 2 cm; beam length L = 0.61 m; beam diameter db = 5.5 mm; plate stiffness h = 2 mm; and aluminum for the material (E = 69 GPa, nu = 0.33).

We start by recalling previous results for this metasurface made of closely spaced beams. It was first introduced in 2014 by^16^, and it has been studied in many ways since: e.g., with numerical simulations, analytical treatments, and experimental observations^3,17–19^. The dominant effect, which is not connected to the spatial configuration of the beams (i.e., ordered vs disordered), is linked to the low quality factor longitudinal resonance of the beams, which creates wide bandgaps and exotic dispersion curves for the first antisymmetric A_0_ Lamb mode propagation. On top of this, if the plate is thin enough, the flexural resonance creates a narrow frequency band perturbation, which affects both S_0_ and A_0_ Lamb modes. Colquitt et al.^19^ proposed an analytical formula for the dispersion curve calculation of such a plate + beam system. In their analysis, the wave propagation inside the metamaterial is governed by a set of equations that involve the two Lamb modes S_0_ and A_0_ in the plate and the two resonances of the beams (longitudinal, flexural). The longitudinal resonance of the beams only interacts with the A_0_ Lamb mode, and the flexural resonance interacts with both A_0_ and S_0_ through coupling terms (Eq. 2.1a-c in^19^). The dispersion equation is obtained in a closed form, but no effective parameters can be expressed, such as the Young’s modulus or the bulk density. On the other hand, the flexibility of the model makes it possible to consider or eliminate the flexural motion of the resonators, which is essential in the proposed multiplexing design. Figure 1 shows dispersion curves computed from Colquitt et al.^19^, with or without the flexural resonance effects (Fig. 1a, b), considering the unit cell parameters defined in Fig. 1c. The A_0_ and S_0_ free plate responses (without the beams) are depicted in gray in Fig. 1a. The bandgap induced by the compressional motion highlighted by the red background color and the blue curve in Fig. 1b includes the beam flexural resonance effects. Figure 1b highlights that the flexural resonances can have different effects according to the frequency of the plate waves in the metamaterial region. In the passband, they interact with the A_0_ wave, but the coupling term is weak and the A_0_ wave dominates. In the bandgap, where A_0_ mode propagation is forbidden, the flexural resonances generated narrow transmission bands, which creates energy leakage from outside to inside the metamaterial (and vice-versa).

In the following, we show that the start of the main bandgap is underpinned solely by the beam length, and the frequency position of the narrow leakage inside the bandgap due to the beam flexural resonance is a function of both the beam length and diameter. Based on this observation, we propose here a passive spatial multiplexer with a clear understanding of the physics, controlling the two types of beam resonances with independent geometrical parameters, i.e., length and diameter. It has the potential for mechanical filtering of the A_0_ Lamb wave in a narrow frequency band and over a wide range of frequencies. Numerical simulations show that the multiplexer waveguides that result have negative refraction indices. Finally, we show that the geometrical periodicity inside of the multiplexing line strongly influences the efficiency of the transmission through the waveguide, highlighting a Fano + Bragg scattering in play.

## Theoretical approach

**Figure 2:**
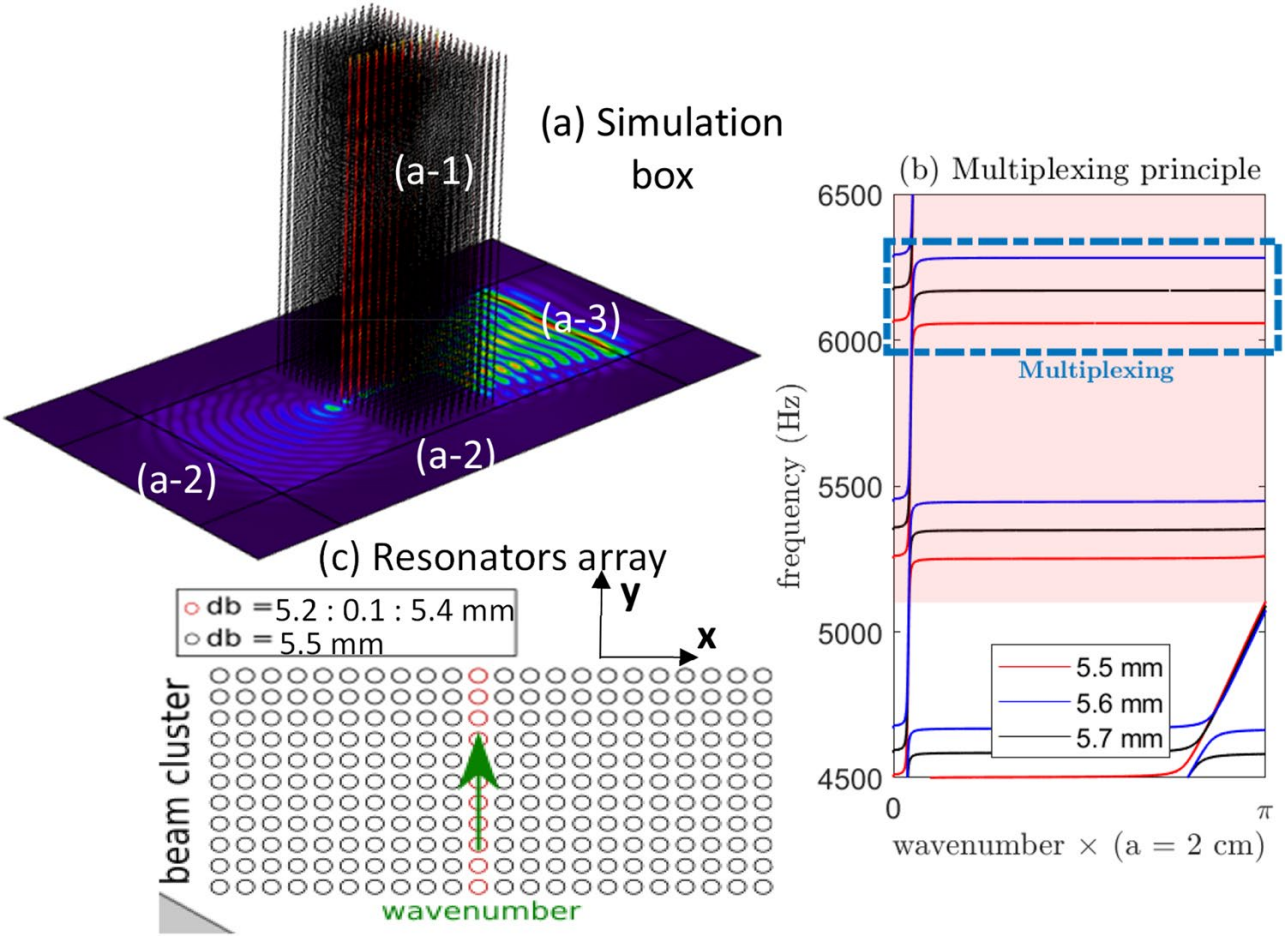
Simulation procedure. (**a**) The metamaterial (**a-1**) is made of 11 × 21 regularly spaced beams attached on a 2-mm-thick plate surrounded by absorbing areas (**a-2**). A plane wave (**a-3**) is emitted from the right side of the beam cluster. (**b**) Typical band structure for three beam diameters. Inside the bandgap (reddish background area), each flexural resonance creates a different narrow leakage (dashed rectangular area). (**c**) Using a different beam diameter along a central line inside the beam cluster tunes the flexural resonance position without affecting the beginning of the bandgap in this region.

Starting with the fully elastic formulation from Colquitt et al.^19^, we estimate the consequences of changing the resonator diameter on the main bandgap and on the narrow flexural leakage, independently. Results of this analysis are presented in Fig. 2b, in a restricted frequency band that includes the end of the passband (~ 5 kHz) and the targeted leakage frequency interval (~ 6 kHz). The main bandgap induced by the compressional motion is highlighted by the red background color (Fig. 2b). The flexural resonances create three distinct narrow bands that leak inside the bandgap, depending on the beam diameter, as highlighted by the dotted square in Fig. 2b. Changing the resonators diameter thus has significant impact on the leakage through the flexural resonance, without affecting the main bandgap.

These results are used here to build a multiplexer, by introduction of local defects into the metamaterial waveguide. These defects are obtained by changing the beam diameter for one line of beams, hence by de-tuning their flexural resonances. In practice, as either longitudinal or flexural, each resonance results in a phase jump at the bottom of the beams. The longitudinal resonance is associated with the up-and-down beam motion that induces a negative apparent density, as seen by the plate^15,20^ with a monopolar radiation into the plate. Similarly, the flexural resonance induces a bending momentum at the bottom of the beams, and thus a dipolar radiation into the plate. The resulting A_0_ scattered field differs substantially, depending on the resonator motion.

The tuned leakage through the metamaterial region represents the association of monopole and dipole resonances, which is crucial to obtain double-negative materials, as is demonstrated herein. In the perspective of further experimental approaches with this device, the geometry constraint here might require a three-dimensional printing technique to create the sample, with a resolution on the geometry construction of less than 0.1 mm.

## Numerical simulations

**Figure 3:**
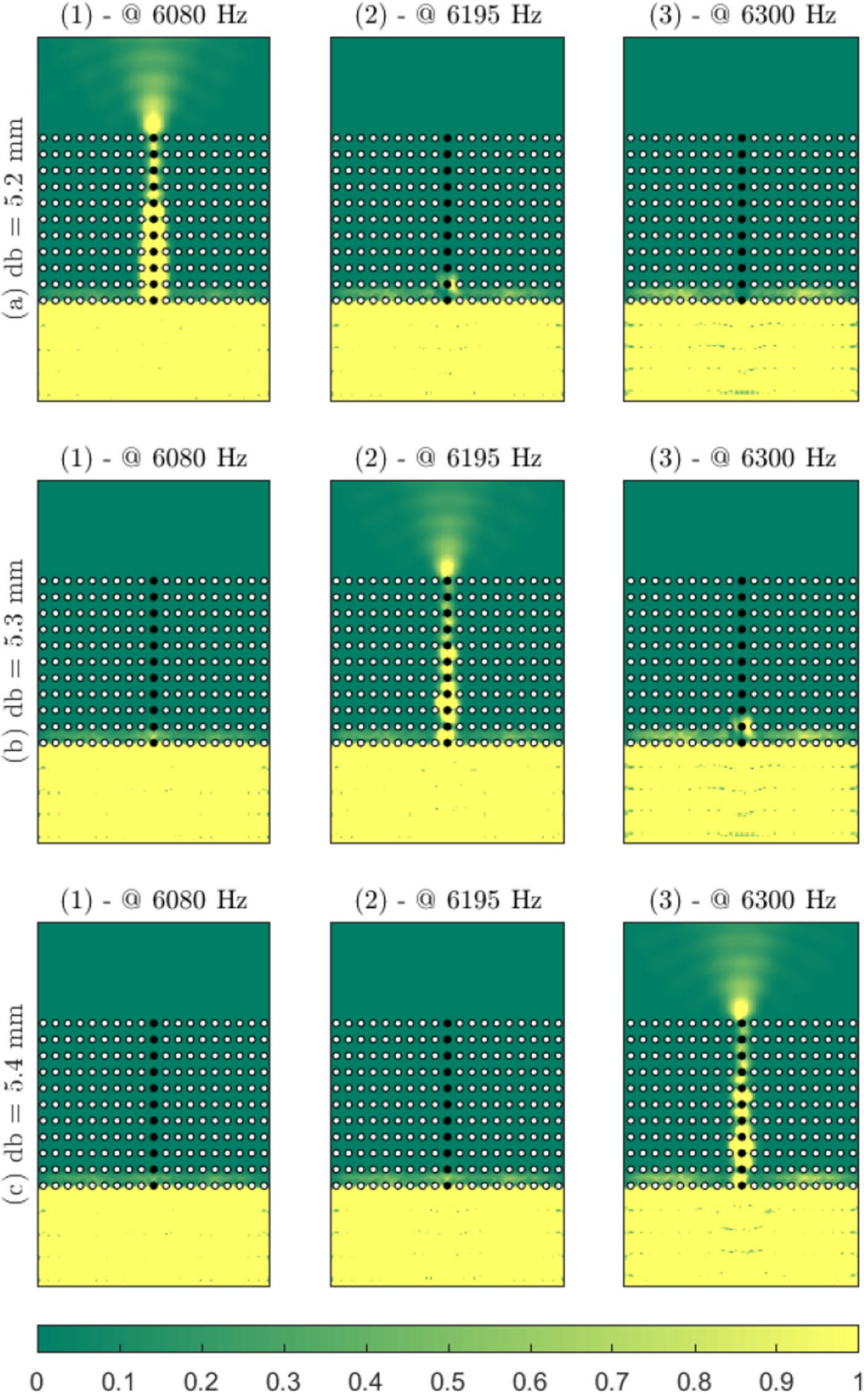
Normalized intensity maps for the three-designed waveguides (normalized by maximum transmitted intensity for each simulation box). The white points represent the 5.5-mm-diameter beams of the cluster, and the black points indicate the 5.2 mm (**a**), the 5.3 mm (**b**), and the 5.4 mm (**c**) diameter beams of the central line. From top to bottom (**a**–**c**): increasing the beam diameter of the central line increases the frequency of the flexural resonance and tunes the leakage through the beam cluster.

We used the COMSOL simulation software to study the propagation of the antisymmetric A_0_ Lamb mode into a metasurface made of 11 × 21 regularly spaced beams. The simulation box is shown in Fig. 2a, which includes the beam cluster (Fig. 2a-1) and the absorbing areas (Fig. 2a-2). The source is a plane wave (Fig. 2a-3) that is emitted from the right side of the metasurface region and transmitted through the beam cluster.

For the propagating simulations, the system is discretized using two-dimensional shell elements to model the plate, and one-dimensional beam elements for the resonators, both of which are available in the structural dynamic toolbox of COMSOL. This strategy greatly decreases the model complexity and the computing time, while preserving the full physics of the system. Using a 2-mm-thick plate in the numerical scheme, both A_0_ and S_0_ are reproduced from 0 to 10 kHz, along with the compressional and flexural motion of the beam. We use the same material and geometry as defined in Fig. 3c. It is now straightforward to introduce local changes in the beam diameter (Fig. 1c), the key parameter in this study, without worrying about meshing instabilities that would arise using full three-dimensional finite elements.

The absorbing boundaries are designed using the approach described in^21^, with eight different areas that surround the model zone that represents the space-dependent attenuation (which increases exponentially from the boundary of the propagating zone to the end of the simulation box). Finally, the full computation takes approximatively 1 h in the frequency domain (around 45 s per frequency). This strategy provides high-frequency resolution in a narrow bandwidth, with limited numerical cost.

## Qualitative and quantitative results

We run three different simulations with varying diameters for the central line of the beam cluster (i.e., the waveguide). The background metamaterial consists of beams with a diameter of 5.5 mm and a length of 61 cm. The central line diameters are 5.2 mm, 5.3 mm, and 5.4 mm for the three simulations. Figure 3 shows the qualitative results of the transmitted intensities here. In the frequency band of 6.0 kHz to 6.3 kHz (Fig. 3, subpanels 1–3), each selected diameter (Fig. 3, subpanels a-c) creates leakage at a very precise frequency, thus realizing a frequency-based selector for the A_0_ Lamb mode.

**Figure 4:**
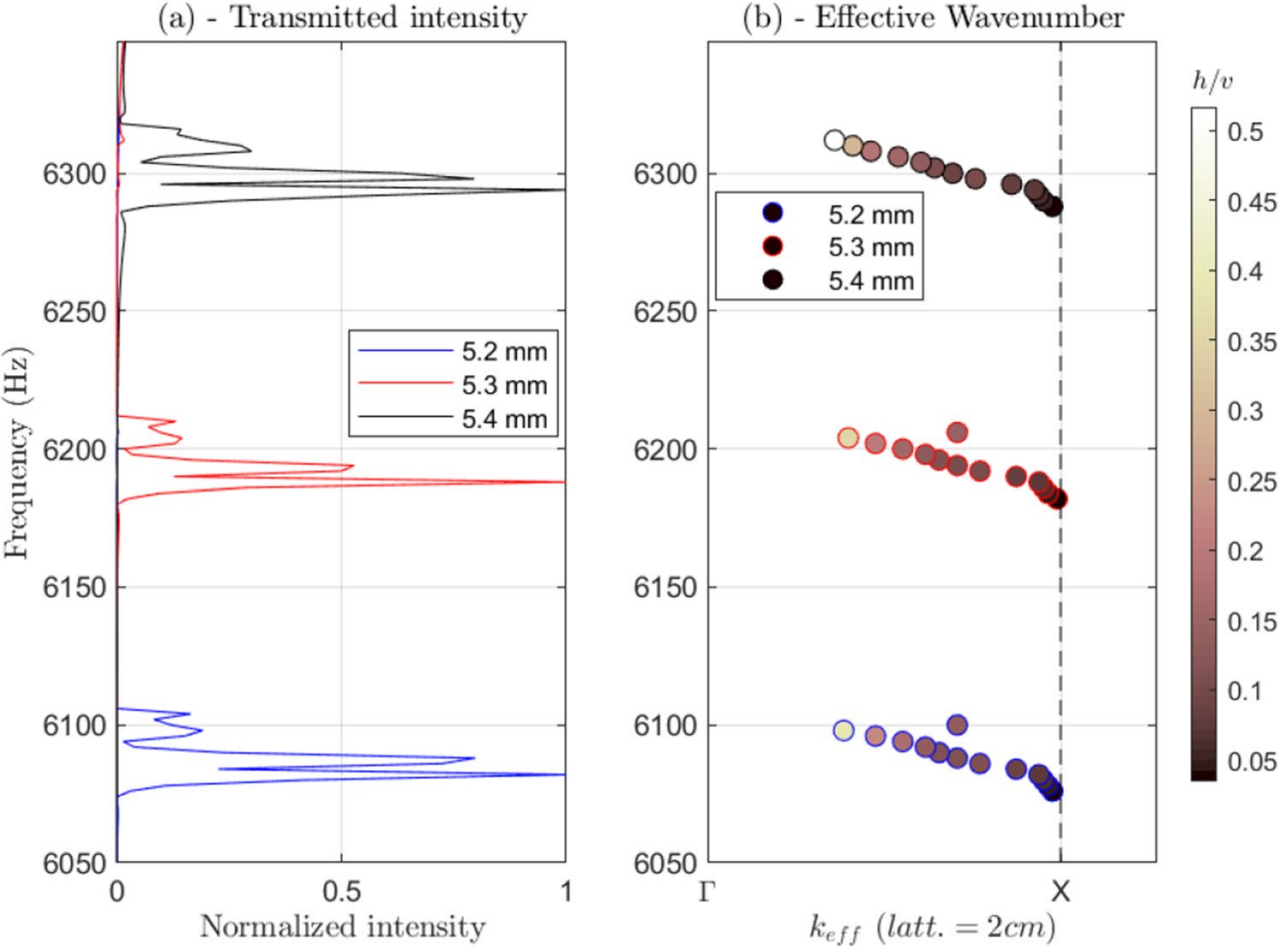
(**a**) Normalized transmission coefficients for the three simulations (blue, red, black) that create three different leakages. (**b**) Band structure of the created waveguides (blue, red, black circles). The color scale in (**b**) depicts the wave polarization through the u/v ratio (i.e., tangential vs. normal components), computed from the spatial Fourier transform.

We also compute the apparent transmission coefficient, as well as the effective wavenumber inside this waveguide. Figure 4 shows the overall results for the normalized transmitted coefficient (Fig. 4a) and the effective wavenumber (Fig. 4b). In Fig. 4a, the three simulations are normalized by the maximum transmitted intensity in the 6.00 kHz to 6.35 kHz band. At around 6.40 kHz (not shown here), the background array made with 5.5-mm-diameter beams globally resonated, which breaks up the wave guidance along the central line. Below this frequency, the three colors in Fig. 4a (blue, red, black) that correspond to the three above-mentioned central line diameters highlight three separate transmission peaks.

For the wavenumber, we simply determine the wave speed along the waveguide. We select three regions to compute the effective wavenumber around each of the transmitted peaks of the three simulations, as plotted in Fig. 4b in an ${\text{f}} - {\text{k}}$ frequency-wavenumber graph. We compute the spatial Fourier transform of the wavefield along the line, and select the propagative wavenumber in the positive y-direction.

The negative slope of the phase speed with respect to frequency can be noted here. With negative slope in the f-k graph, and thus negative group velocity, the double negativity typical response of this metamaterial is highlighted. Due to the periodicity of the designed array, we display the calculation of the effective wavenumber on the Brillouin edges ($\Gamma -\mathrm{X}$) (Fig. 4b). However, we do not observe any spectral folding after ‘X’ here. Note that the $\Gamma  - {\text{X}}$ direction (Fig. 4b) corresponds to the reciprocal space ‘y’ direction in Fig. 2c.

Also, along with these three detected bands, we compute the spatial–spectral amplitude of the tangential and normal components of the wavefield that propagates along the positive y-direction, for the previously estimated wavenumber. We estimate the horizontal *versus* vertical motion of the plate through the spatial Fourier transform of both in-plan (h) and out of plane (v) motion of the plate surface. The values obtained are reported in the color scale in Fig. 4b.With the ratio u/v (i.e., the tangential vs. vertical components), we observe smooth transition between ‘quasi’ tangential waves and ‘quasi’ normal ones. This transition was already expected by Rupin et al.^22^, who also described the coupling between the two orthogonal Lamb modes of the free plate (A_0_-S_0_) in this frequency regime.

## Discussion

Previous analytical studies of such a plate + beam system have highlighted the dependency between the slope of the quasi-flat band induced by the flexural resonance inside the bandgap and the overall geometric properties of the system. In particular, the thickness of the plate substrate^19^ influences the emergence of a negative index transmission band. Indeed, if the plate is thin enough, the bending moment induced by the flexural resonance of the beams can add negative mass density, in addition to the negative Young’s modulus induced by the longitudinal resonance inside the bandgap [Williams et al. 2016], and thus yield a negative group velocity^23^.

In practical instances, double-negative materials come with high attenuation effects^10^. Here, due to the finite size of the system, our transmission coefficient calculation does not capture quantitatively the reflection magnitude at the plate/metamaterial interface. However, previous experimental and numerical data have demonstrated that leakage of such a flexural resonance inside the wide bandgap induced by the compressional motion of the beams can be detected easily^22,24^. In the present study, we move our attention to the propagation mechanism along the defect line through the introduction of progressive disorder in the multiplexer geometry, to thus identify the scattering regime that is in play. The results of these simulations are shown in Fig. 4.

**Figure 5:**
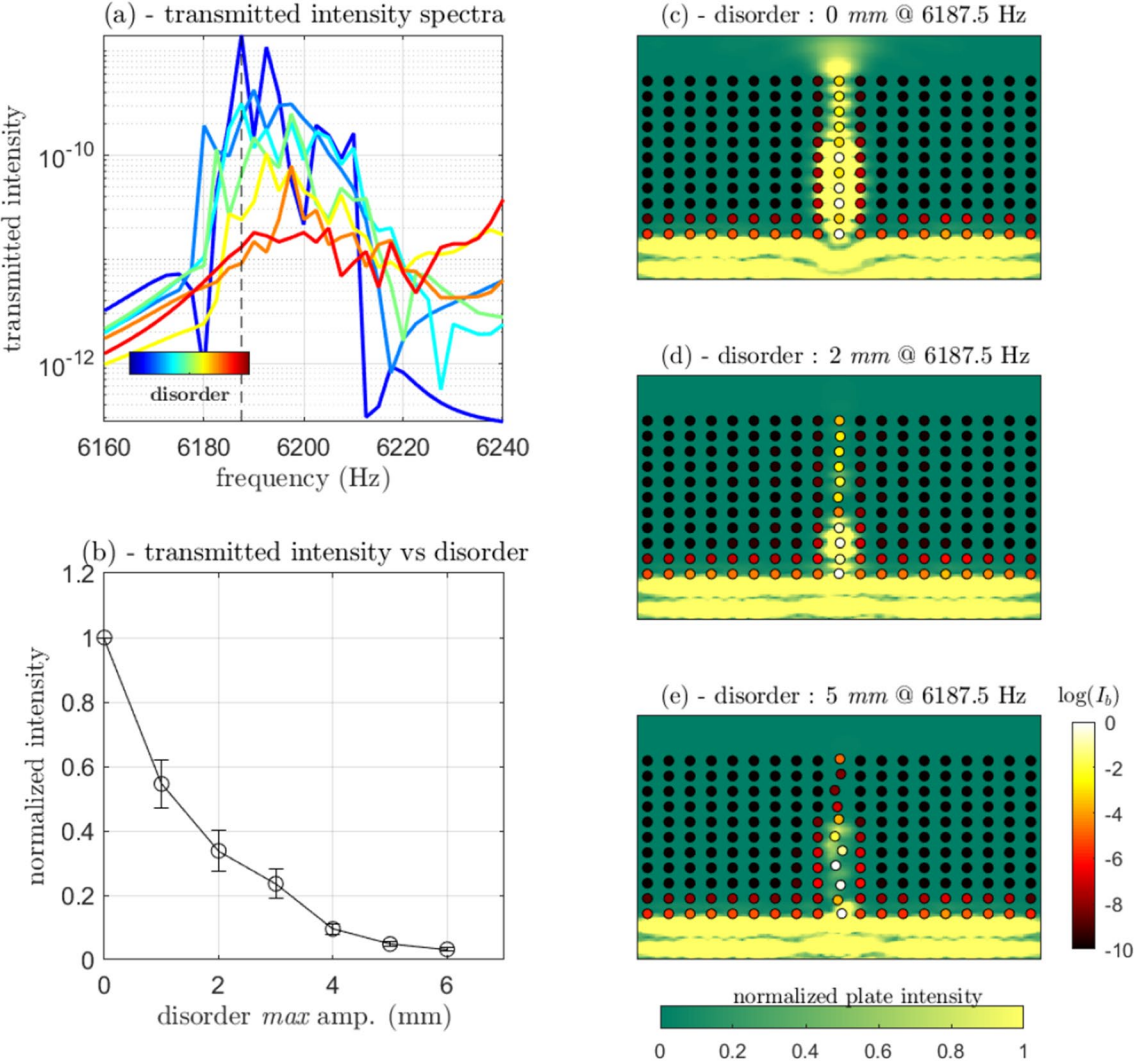
Effects of the randomness of the central line beam positions on the transmission intensity. (**a**) Mean transmission spectra for the 5.3-mm-diameter beams in the central line and the different values of the disorder. (**b**) Normalized transmitted intensity versus amplitude of the disorder, as indicated. (**c**) same as Fig. 2b at 6195 Hz. (**d**, **e**) Intensity map at 6195 Hz for 2 mm and 5 mm random displacements of the beams in the central line. The intensity maps are normalized by the maximum transmitted intensity for the case without disorder. (**c**–**e**) The beam motion amplitude is depicted as a ‘log’ scale.

The numerical model is similar to that shown in Fig. 3b. The disorder is implemented by induction of a small spatial random variation along the waveguide (0–6 mm, drawing from a uniform distribution). For each disorder value, three simulations were run, with calculation of the mean transmitted intensity spectra. The results of these spectra are shown in Fig. 5a. With disorder, the transmission peak decreases in amplitude. The mean transmitted intensity integrated on the full frequency band (6160–6240 Hz) is shown in Fig. 5b as a function of the disorder, where the error bars represent the standard deviations of the simulation results for each disorder value. It is worth noting that the fluctuations over disorder are stronger at a single frequency than in the integrated frequency band.

Figure 5c-e (Fig. 5c is the same as Fig. 3b, but at 6195 Hz) shows the intensity map at 6195 Hz for three particular values of the disorder (i.e., 0, 2, 5 mm random displacement of the beams). In Fig. 5c, the beams are depicted as small circles with a black-to-white color scale as the logarithm of the beam motion intensity, and a green-to-yellow color scale as the normalized transmitted intensity (the norm is the maximal transmitted intensity with 0 disorder, as in Fig. 3). We observe that with increasing disorder, the intensity diffusion across the central line might stop. This result differs from previous experimental studies that have shown the non-effect of the randomness of the beams positions on the main A_0_ passband^16^. Here, the spatial ordering is essential in this propagative branch, making the coupling between the S_0_ and A_0_ Lamb modes possible, from the tangential force for the low spatial frequency regime (low k values), to the predominance of the bending motion for the high spatial frequencies (high k values). We conclude that the propagation in this frequency band is due to Bragg dipolar scattering between successive aligned beams, which is highly sensitive to disorder. In the absence of disorder, the combination met the criteria for a one dimensional negative index material, resulting in the negative slope in the f-k representation of Fig. 4b.

## Conclusion

We demonstrate here the possibility to model a device that can filter and guide low-frequency Lamb waves in a thin plate using the modal overlap of the flexural and compressional resonances of the beam-like resonators. The frequency position of the flexural resonance where the leakage is observed can be adjusted by acting on the diameter of one line of beams, which does not affect the longitudinal resonance that controls the main bandgap. Building on these results, we model a mechanical wave multiplexer that can select the narrow frequency flexural Lamb mode inside a wide frequency bandgap.

With the combination of two scattering modes, one monopolar and one dipolar, the resulting effective material has a negative refraction index with fast evolution of wave polarization over frequency. The effects of the randomness of the beam positions on the waveguide efficiency are also evaluated, and these confirm the predominance of a Bragg scattering mechanism of intensity diffusion for the flexural resonance of the beams, in addition to the mechanical constraints at the beam attachment due to compressional resonance inside the main bandgap. These results highlight the strong interplay between hybridization due to local resonance, hybridization between different resonant modes, and Bragg scattering versus incoherent scattering. We believe these results can be adapted to any locally resonant system if the individual resonators overlap in their Fano resonances.
